# Within-subject reliability, occasion specificity, and validity of fluctuations of the Stroop and go/no-go tasks in ecological momentary assessment

**DOI:** 10.3758/s13428-024-02567-1

**Published:** 2024-12-28

**Authors:** Justin Hachenberger, Axel Mayer, Denny Kerkhoff, Friederike Eyssel, Stefan Fries, Tina B. Lonsdorf, Hilmar Zech, Lorenz Deserno, Sakari Lemola

**Affiliations:** 1 Department of Psychology, Bielefeld University, Universitätsstraße 25, 33615 Bielefeld, Germany; 2 Center for Cognitive Interaction Technology (CITEC), Bielefeld University, Bielefeld, Germany; 3 Department of Systems Neuroscience, University Medical Center Hamburg-Eppendorf, Hamburg, Germany; 4 Department of Psychiatry and Psychotherapy, Technische Universität Dresden, Dresden, Germany; 5 Department of Child and Adolescent Psychiatry, Psychotherapy and Psychosomatics, Center for Mental Health, University Hospital Würzburg, Würzburg, Germany

**Keywords:** Cognitive control, Stroop, Go/no-go, Ecological momentary assessment, Dynamic structural equation modeling, Intensive longitudinal design

## Abstract

Following the (revised) latent state–trait theory, the present study investigates the within-subject reliability, occasion specificity, common consistency, and construct validity of cognitive control measures in an intensive longitudinal design. These indices were calculated applying dynamic structural equation modeling while accounting for autoregressive effects and trait change. In two studies, participants completed two cognitive control tasks (Stroop and go/no-go) and answered questions about goal pursuit, self-control, executive functions, and situational aspects, multiple times per day. The sample (aged 18–30 years in both studies) consisted of 21 participants (14 female) in the pilot study and 70 participants (48 female) in the main study. Findings indicated poor within-subject reliability for the Stroop task error rate and reaction time difference between congruent and incongruent trials and moderate to good within-subject reliability for the go/no-go task error rate and reaction time. Occasion specificity—the systematic variance accounted for by state residuals—was at a modest level (between 1.4% and 11.1%) for the Stroop error rate and reaction time difference, and at a moderate level (between 16.1% and 37.2% for the go/no-go error rate and reaction time) in the two studies. Common consistency—the variance accounted for by latent trait variables—was at a moderate to high level for all of the investigated scores. Indicative of construct validity, the Stroop and go/no-go task error rates correlated positively with each other on the within- and between-subject level. Within-subject correlations between task scores and subjective self-control measures were very small and mostly nonsignificant.

## Introduction

The ability to apply cognitive control over one’s actions and to inhibit dominant responses has typically been measured with cognitive control tasks such as the Stroop task (MacLeod, 1991) or the go/no-go task (Falkenstein et al., 1999). Recently, researchers have started to embed cognitive control tasks into the context of ecological momentary assessment (EMA) to measure either whether fluctuations in cognitive control are predicted by fluctuations in intrapsychic or contextual variables, or whether cognitive control predicts behavioral outcomes (e.g., Zech et al., 2023). First studies indicate that fluctuations in cognitive control measured by EMA can predict changes in real-world behavior. For example, Powell et al. (2017) showed that intra-individual fluctuations in the performance on the go/no-go task predicted subsequent snacking behavior in within-subject analyses, whereas no associations of individuals’ mean performance and snacking behavior were observed in between-subject analyses. Moreover, better state-level performance on the go/no-go task was associated with reduced negative emotionality (Rónai et al., 2024) and better mood specifically in individuals with higher trait levels of resilience (Nahum et al., 2023), indicating the potential importance of within-individual fluctuations in cognitive control for positive functioning and real-world behavior. Similarly, within-individual fluctuations in the reaction time measured with the stop-signal task predicted fluctuations in alcohol consumption in heavy drinkers (Jones et al., 2018). Thus, within-individual fluctuations in cognitive control tasks appear to predict within-individual fluctuations in real-world behavior.

Despite the growing interest in within-individual fluctuations in cognitive control in the ecological context, existing research on the reliability and validity of cognitive control tasks has mainly focused on the laboratory context. Using only a limited number of measurement occasions, this laboratory-based research has shown moderate to excellent levels of (overall) reliability and modest to moderate correlations between different cognitive control tasks, depending on the specific tasks studied (see, e.g., Faßbender et al., 2023). While research in the EMA context is still scarce, existing studies on the reliability and validity of a cognitive control task (i.e., the stop-signal reaction time) in the intensive longitudinal design indicated moderate levels of retest reliability and moderately high factor loadings on a common executive control factor (Zech et al., 2022; Zech et al., 2023). Surprisingly, however, existing research on cognitive control measures in EMA has to our knowledge not reported within-subject variability measures such as within-subject reliability and occasion specificity. The former—within-subject reliability—quantifies the reliability of the measurement of within-person fluctuations. It is also referred to as the “reliability of change” (Neubauer & Schmiedek, 2020) or the measurement of shared “ups and downs” of indicators across time (Brose et al., 2020). In contrast, the latter—occasion specificity—measures the proportion of overall variance in the response that is due to systematic situational effects, including situation–person interactions.

Specifically, occasion specificity is defined by the revised latent state–trait theory (LST-R; Steyer et al., 2015), an extension of classical test theory. According to LST-R, total variance can be decomposed into “true variance” and “error variance,” and true variance can be further decomposed into “trait-level variance” and “state residual variance.” The proportions of trait-level variance and state residual variance in the total variance are defined as common consistency and occasion specificity, respectively. While common consistency is assumed to be mainly due to genetic and long-standing environmental influences as well as gene–environment interactions (Plomin et al., 1977), occasion specificity is thought to be impacted by situational influences and person-by-situation interactions (Steyer et al., 1999). Thus, both within-person reliability and occasion specificity quantify aspects of the measurement of within-person fluctuations. However, they measure distinct components: While within-person reliability measures the reliability of a set of items to capture systematic fluctuations within persons and across time (i.e., measurement occasions) specifically in relation to the within-person variance, occasion specificity measures the proportion of systematic within-person variance (i.e., situational effects and person-situation interactions) in relation to the total response variance. Reporting of these indices is important for estimating the statistical power of a study design to detect systematic within-subject variability in these measures and thus to determine whether a study had a realistic chance of detecting within-subject associations between fluctuations in cognitive control measures and fluctuations in other variables including situational predictors and behavioral outcomes.

Indeed, fluctuations in cognitive control have been shown to be influenced by several situational variables including concurrent stress levels (Shields et al., 2016), positive and negative affect (Dreisbach, 2006), motivation (Botvinick & Braver, 2015; Inzlicht & Berkman, 2015), mental exhaustion (Inzlicht & Berkman, 2015), arousal levels (Lo et al., 2016), acute physical activity and exercise (Ludyga et al., 2016), circadian timing (Blatter & Cajochen, 2007), sleep duration of the previous night (van Dongen et al., 2003; Lim & Dinges, 2010), substance use and intoxication (McPhee & Hendershot, 2023), and situational distraction such as by noise (Szalma & Hancock, 2011). Reporting of the LST-R-indices reliability, occasion specificity, and common consistency of cognitive control measures is essential for understanding the degree to which test scores are due to measurement error, situation-specific influences, and stable person characteristics.

Similar to within-subject reliability, occasion specificity is of particular importance for understanding how much variance of an intensive longitudinal study is due to systematic fluctuations. Previous studies that aimed to quantify occasion specificity of cognitive control, however, have shown only a small proportion of variance being explained by the occasion compared to a much larger trait component. Faßbender et al. (2023), for instance, using a laboratory setting, only found 2% of the total variance explained by occasion specificity in the Stroop task congruent–incongruent reaction time (RT) differences, while common consistency (i.e., trait component) was estimated at 50%. For the go/no-go task error rate (ER), 6% of the variance was explained by occasion specificity, while the common consistency explained 55%. Similar estimates were found by Meyhöfer et al. (2016). However, an important limitation of these two studies is that they measured cognitive control in highly standardized laboratory settings to minimize occasion-specific influences. For instance, they only used three measurement time points in total, which were all scheduled at the same time of day and without occasion-specific experimental manipulations. While such procedures are adequate for measuring peak performance and increasing the amount of variance explained by the trait component, thereby leading to higher retest reliability, laboratory settings naturally lack ecological validity. In contrast, cognitive control tasks embedded in EMA may provide higher levels of ecological validity (Zech et al., 2023). Moreover, a more even distribution of occasion-specific variance and variance due to common consistency may be expected if the number of measurement occasions is similar to the number of participants in the study.

One EMA study that investigated within-subject reliability for other cognitive tasks including working memory tasks (i.e., N-back and dot memory task) and processing speed (i.e., symbol search task) showed moderate levels of within-subject reliability (Sliwinski et al., 2018). Sliwinski and colleagues also examined whether within-subject reliability changed across the duration of their EMA study, as it is possible that with increasing length of the EMA, study participants’ motivation decreases, which might negatively impact reliability. On the other hand, it is also possible that reliability is lower in the early phase of the study when participants are not as familiar with the tasks as later on. Importantly, within-subject reliability of working memory and processing speed was not associated with the duration of the EMA protocol, which was shown by a constant level of within-subject reliability across 14 study days.

### The present study

The present studies had the following four goals.

First, we aimed to establish within-subject reliability alongside the core indices of LST-R including measurement reliability, occasion specificity, and common consistency of two commonly used cognitive control/inhibition tasks, the Stroop and the go/no-go task, in the EMA context. Our specific interest here, apart from within-subject reliability, is focused on occasion specificity. The within-subject reliability and LST-R indices were estimated by dynamic structural equation models (Asparouhov et al., 2018; Castro-Alvarez et al., 2022; Geiser et al., 2013), which allowed us to account for effects of inertia by modeling auto-regressions between adjacent measurement occasions. Moreover, endorsing the possibility of trait change across the duration of the EMA study as envisaged by the revision of LST theory (Steyer et al., 2015), we additionally modeled a linear trend of the trait variable as a fixed effect. It is conceivable that the trait levels of the task performance may change over the course of the intensive longitudinal study, due to learning experience (which would result in an improvement in task performance over time) or fatigue and/or boredom (which would result in a decrease in task performance over time).

Second, we aimed to estimate the number of test trials of the Stroop and go/no-go task necessary to achieve an acceptable degree of within-subject reliability. Following Sliwinski et al. (2018), we also aimed to establish whether within-subject reliability changed across the duration of the EMA protocol.

Third, we aimed to estimate the convergent validity of the Stroop and go/no-go tasks while disentangling between-subject (trait) and within-subject (state) levels. For this we calculated between- and within-subject Pearson correlations. To estimate the convergent validity, the correlations between the Stroop and go/no-go tasks were used, which both measure the ability to inhibit a dominant response tendency (Miyake et al., 2000). We hypothesized that we would find positive correlations in particular at the within-subject level between the Stroop and go/no-go task ERs, which conceptually best represent the (un)successful inhibition of a dominant response (whereas e.g. the go/no-go task RT rather reflects processing speed, as only RTs in go trials are considered, which is conceptually more closely related to Stroop RTs in congruent trials). Furthermore, the hypothesis is focused on the within-subject association for which the detection is facilitated due to higher statistical power. Previous research has only shown cross-sectional associations between Stroop and go/no-go indices (Lamm et al., 2006; Morooka et al., 2012), and the Stroop RT in particular was not correlated with the go/no-go ER at the between-subject level in one study with repeated measures (Faßbender et al., 2023). Furthermore, the two cognitive control tasks were correlated with self-reported measures of executive functions, self-control, and goal pursuit, which were also measured in EMA and were expected to correlate with cognitive control tasks to a more modest degree (Paap et al., 2020).

Fourth, we examined how much of the within-subject variance in cognitive control tasks was explained by common contextual variables in the EMA context including time of day, location (indoors vs. outdoors), social context (being together with other people vs. alone), and self-reported tiredness. To reach these four goals we conducted two EMA studies employing an iterative approach. We first conducted a pilot study to inform adaptations to the procedure of a larger main study.

## Method

### Procedure

We conducted a pilot study and a main study in accordance with the Declaration of Helsinki and with approval of the Ethics Committee of Bielefeld University (reference number 2023-160). Initially, participants were informed about the procedure and conditions of participation as well as their rights. All participants provided informed consent and confirmed that they fulfilled prerequisite criteria. German native speakers without dyschromatopsia, aged 18 to 30 years, and who had a smartphone with an Android operating system available for the duration of the study were eligible to participate in the research.

The data collection lasted 15 days (one day baseline assessment, 14 days EMA) and took place in July 2023 (pilot study) and August/September 2023 (main study). On the first day, the participants completed a baseline questionnaire. On the second day, EMA questionnaires and cognitive tasks (Stroop, go/no-go) started. The EMA questionnaires were presented in movisensXS (version 1.5.23; library version 8399; movisens GmbH, Karlsruhe, Germany). The cognitive tasks were presented using Presentation for Android (version 3.0.5, Neurobehavioral Systems, Inc., Berkeley, CA, USA), but were initiated within the experience sampling questionnaires. Participants received five prompts per day in the pilot study and four prompts per day in the main study to complete a short questionnaire and the cognitive tasks for 14 consecutive days. Each prompt had the following sequence: (1) completing the questionnaires, (2) performing the Stroop task (two blocks), and (3) performing the go/no-go task (two blocks). In the main study, participants additionally performed the fruit tapping task (a gamified version of the stop-signal task; Smittenaar et al., 2015; Zech et al., 2023), which is not part of the current study.

In the pilot study, participants were able to manually start the first questionnaire of each day and were asked to do so as soon as possible after waking up. If they did not start the first questionnaire manually, participants received a reminder prompt between 8.30 a.m. and 9.30 a.m. For the next four questionnaires during the day, a prompt was sent at random times within the following time intervals: (1) 10.30–11.30 a.m., (2) 2.00–3.00 p.m., (3) 5.30–6.30 p.m., and (4) 8.30–9.30 p.m. In the main study, participants received prompts for the four questionnaires of a day at random times within the following time intervals: (1) 9.30–10.30 a.m., (2) 1.00–2.00 p.m., (3) 4.30–5.30 p.m., and (4) 8.00–9.00 p.m.

In both studies, participants could respond to each questionnaire within 30 minutes after receiving each prompt. If participants did not respond within 30 minutes, this questionnaire was marked as missing. Participants were instructed to ignore prompts in situations that could endanger themselves or others (e.g., while driving).

### Participants

Participants were recruited via email lists for study advertisements, the study participation management system of the Department of Psychology at Bielefeld University, and word-of-mouth advertisements among the students.

In the pilot study, the sample consisted of 21 participants (66.7% female, 33.3% male; mean age = 25.1 years, *SD* = 3.4) providing 986 assessments (mean per person = 47.0, *SD* = 21.3) after the exclusion criteria were applied (see “Data Preprocessing”). Participants who completed at least 80% of all assessments received a €60 voucher. Participants who completed at least 50% but less than 80% of all assessments received a €30 voucher. Three €100 vouchers were raffled among all participants qualifying for compensation.

In the main study, the sample consisted of 70 participants (68.6% female, 25.7% male, 5.7% preferred not to answer; mean age = 24.7 years, *SD* = 3.4) providing 2481 assessments (mean per person = 35.4, *SD* = 18.1) after exclusion criteria were applied (see “Data Preprocessing”). Participants who completed at least 50% of all assessments were eligible to receive compensation. The amount of compensation depended on the number of completed assessments, with €2.50 per assessment.

### Measures and instruments

#### Color Stroop task

**Fig. 1:**
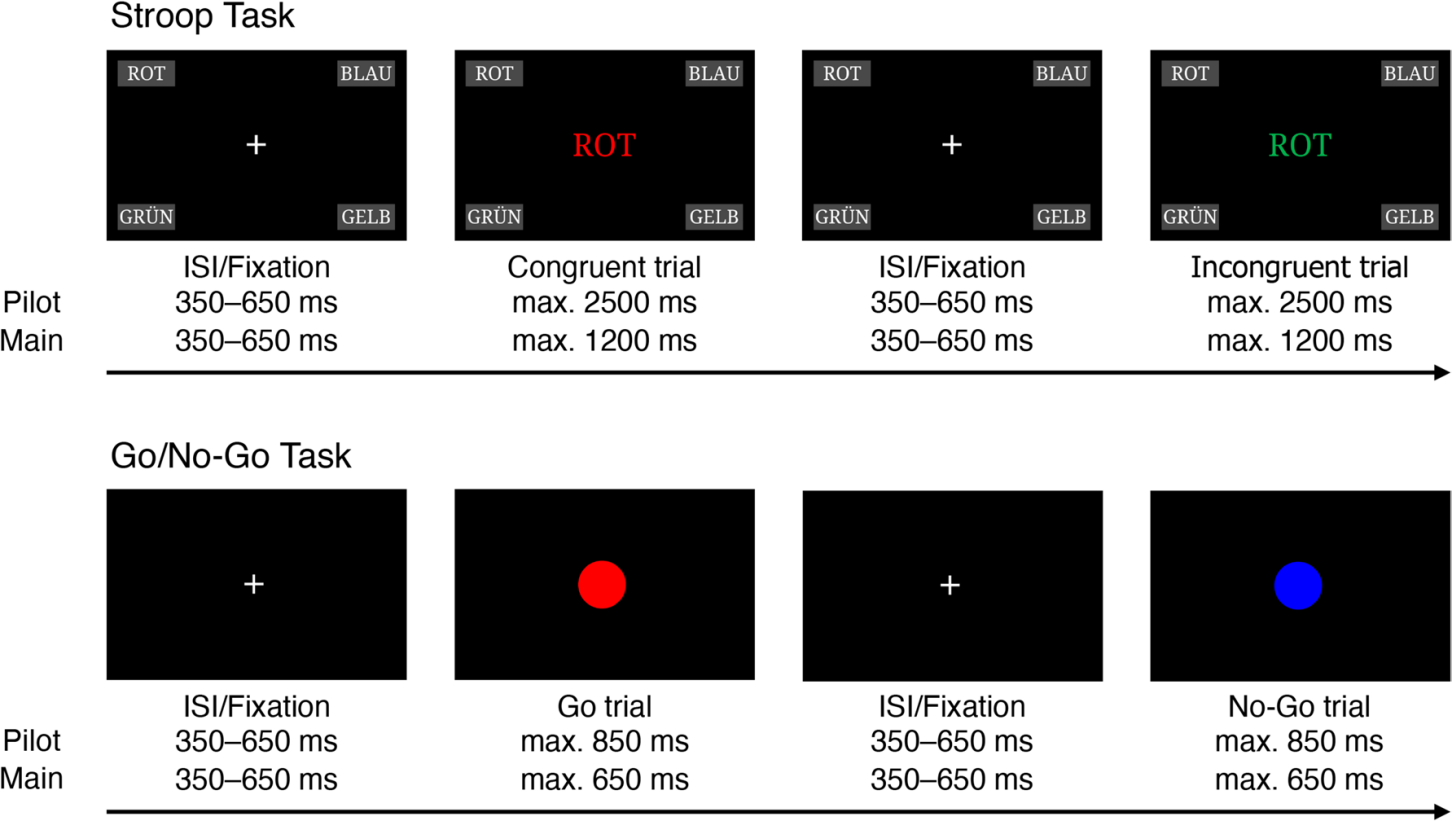
Schematic of the Stroop and go/no-go tasks. *Note*. *ISI* interstimulus interval

Each trial of the Stroop task started with a central fixation cross presented for a random duration between 350 and 650 ms on black background, followed by a stimulus presentation of the German word for red (“rot”), blue (“blau”), green (“grün”), and yellow (“gelb”). The words were printed in red (RGB 255, 0, 0), blue (RGB 0, 0, 255), green (RGB 0, 255, 0), or yellow (RGB 255, 255, 0). In each trial, the stimulus was presented until a response was given or for a maximum of 2500 ms in the pilot study and 1200 ms in the main study. A response could be given via four buttons placed in the corners of the smartphone display. Each button had a gray background, and the corresponding response option was in white font (top left: “rot”, top right: “blau”, bottom left: “grün”, bottom right: “gelb”). Participants were instructed to tap the button that referred to the same color as the stimulus word was printed in and not to the meaning of the word. In each session, participants were instructed to hold the smartphone horizontally with both hands so that they could use both thumbs to respond. Participants were asked to respond as quickly and accurately as possible. The stimuli could be categorized as congruent (e.g., “rot” displayed in red font color) or incongruent (e.g., “rot” displayed in green font color). In the pilot study, the Stroop task consisted of two blocks of 12 congruent trials (3 × each congruent combination) and 12 incongruent trials (1 × each incongruent combination) in randomized order (i.e., 48 trials in total). In the main study, the task consisted of two blocks of 24 congruent trials (6 × each congruent combination) and 24 incongruent trials (1 × each incongruent combination) in randomized order (i.e., 96 trials in total). In both studies, each block started with four additional congruent practice trials (fixed order: red, blue, green, yellow) so that the participants were reminded of the button positions and could memorize them. These four practice trials were not considered for analysis. A schematic of the Stroop task is displayed in Fig. 1.

#### Go/no-go task

In each session, participants were first instructed to hold the smartphone horizontally with both hands and to use the thumb of their dominant hand to respond. Participants were asked to tap the screen (any position on the screen) when a red circle (i.e., go stimulus; RGB 255, 0, 0) appeared and contain their response when a blue circle (i.e., no-go stimulus; RGB 0, 0, 255) appeared. In the pilot study, the task consisted of two blocks of 28 go stimuli and 12 no-go stimuli in randomized order (i.e., 80 trials in total, with 30% no-go stimuli). In the main study, the task consisted of two blocks of 80 go stimuli and 20 no-go stimuli in randomized order (i.e., 200 trials in total, with 20% no-go stimuli). The go stimuli were presented more frequently than the no-go stimuli so that a tendency to respond to go stimuli was elicited. Each trial consisted of a central white fixation cross presented for a random duration between 350 and 650 ms followed by a stimulus presentation until a response was given or for a maximum of 850 ms in the pilot study and 650 ms in the main study. A schema of the go/no-go task is displayed in Fig. 1.

#### Executive function problems

To assess executive function problems, an adapted version of the Webexec (Buchanan et al., 2010) was used. The six items of the Webexec were adapted so that participants were asked in each questionnaire about problems within the last 30 minutes in different aspects of executive function (keeping attention focused on a particular task; concentrating on a task; carrying out more than one task at a time; losing one’s train of thought; seeing tasks through to completion; controlling impulsivity). Participants could answer each item on a visual analogue scale ranging from 0 (no problems experienced) to 100 (severe problems experienced). An overall score was calculated by summing the scores of all items, which showed excellent between-subject reliability (Ω_between_ = .98) and within-subject reliability (Ω_within_ = .90) in the pilot study and excellent between-subject reliability (Ω_between_ = .97) and within-subject reliability (Ω_within_ = .90) in the main study.

#### Self-reported goal pursuit and self-control measures

Participants were asked whether they had been busy pursuing a relevant goal in the last 30 minutes (“Have you been busy pursuing a relevant goal in the last 30 minutes?”). If yes, participants could also indicate how much progress they had made towards their goal (“In the last 30 minutes I have achieved this goal or made good progress towards it.”) on a visual analogue scale ranging from 0 (no progress) to 100 (very great progress).

Based on items that were adapted from Hofmann et al. (2012a, b), participants were also asked whether they were currently experiencing a desire or had experienced a desire in the last 30 minutes (“Are you currently experiencing a desire or have you experienced a desire in the last 30 minutes?”). If so, participants were also asked how strong this desire was (“How strong is/was this desire?”), to what extent it conflicted with current goals (“To what extent does this desire conflict with your current goals?”), and how well they were able to resist the desire (“In the last 30 minutes, I was able to resist this desire well.”). Participants could respond to each item on a visual analogue scale from 0 (not at all) to 100 (very much).

Based on items validated by Wolff et al. (2022) that were adapted to the EMA context, participants were asked how self-disciplined they were in the last 30 minutes (“In the last 30 minutes, I was self-disciplined.”) and to what extent they exerted willpower to achieve their goal (“In the last 30 minutes, I exerted willpower to stay focused on my goals.”). Participants could respond to each item on a visual analogue scale from 0 (not at all) to 100 (very much). A sum score of both items was computed for further analysis, which showed good between-subject reliability (Ω_between_ = .89) and good within-subject reliability (Ω_within_ = .80) in the pilot study and good between-subject reliability (Ω_between_ = .79) and good within-subject reliability (Ω_within_ = .79) in the main study. To also assess a situational strategy of self-control, which according to the process model of self-control (Duckworth et al., 2016) plays an important role in preventing temptations from arising and thus decreasing the need to exert cognitive control related to suppression of thoughts or emotions, participants were also asked to what extent they made sure that their environment was conducive to achieving their goals (“I made sure that my environment was conducive to focused work (or to achieving my goals).”). Participants could respond to each item on a visual analogue scale from 0 (not at all) to 100 (very much).

#### Contextual variables

The current social context was assessed by asking the participants to indicate who they were currently with (“Who are you currently with?”), with multiple options. For further analysis, responses were classified into “being alone” and “being with others.”

The current location was assessed by asking the participants where they were (“What is your current location?”), with multiple options. For further analysis, responses were classified into “being inside” and “being outside.”

Tiredness was assessed by asking the participants to what extent they currently felt tired (“How tired do you feel at the moment?”). Participants responded on a visual analogue scale ranging from 0 (not at all) to 100 (very much).

### Data preprocessing

Data preprocessing and all statistical analyses were performed using R (R Core Team, 2023) and Mplus (Muthén & Muthén, 1998–2017).

#### Task scoring

When scoring the Stroop and go/no-go task, we focused on indices that were frequently used in the literature for the two cognitive control tasks (see Faßbender et al., 2023). For the Stroop task, we calculated the error rate in incongruent trials (hereafter referred to as Stroop ER) and the reaction time difference between congruent and incongruent trials (hereafter referred to as Stroop task RT-Diff). For the go/no-go task, we calculated the error rate in no-go trials (hereafter referred to as go/no-go ER) and the reaction time in go trials (hereafter referred to as go/no-go RT). Before calculating the scores, we applied restrictions following Faßbender et al. (2023): (1) Trials with RT < 150 ms were marked as invalid, (2) RT scores were only based on correct responses, and (3) at least ten trials per block and condition (i.e., “congruent” and “incongruent” in the Stroop task and “go” and “no-go” in the go/no-go task) had to be available to be considered for scoring. In order to allow for decomposition of within-subject and between-subject variance, both Stroop and go/no-go tasks were measured in two separate blocks within each measurement occasion, and from each block one indicator/aggregate was derived.

#### Exclusion criteria

We applied several exclusion approaches to account for careless responding that could bias the statistical analyses: (1) For each prompt, we calculated the time the participants needed to complete the questionnaire which was filled out before completing the tasks. If the average processing time per item was < 1 second, the respective prompt was excluded from further analyses (Jaso et al., 2022). (2) For the Stroop task, we calculated an index indicating the maximum sequence of subsequent identical responses per block using the R package *careless* (Yentes & Wilhelm, 2023). Prompts with an index of more than three standard deviations above the mean were also excluded from further analysis. (3) If the error rate in one of the two tasks was three standard deviations above the mean, the scores for the respective task were excluded from further analyses. Based on these criteria, a total of seven occasions of the Stroop task and eight occasions of the go/no-go task were excluded from the pilot study. In the main study, a total of 54 occasions of the Stroop task and 55 occasions of the go/no-go task were excluded.

### Statistical analysis

#### (Overall) reliability, common consistency, occasion specificity, and within-subject reliability estimated using dynamic structural equation modeling (DSEM)

To establish reliability, occasion specificity, and common consistency while accounting for trait and state changes over time, we computed first-order autoregressive [AR(1)] DSEM models in Mplus (Asparouhov et al., 2018; Muthén & Muthén, 1998–2017). The data and analysis code can be found on the Open Science Framework (https://osf.io/rjbx2/). Only participants with at least five complete assessments were considered. Separate but structurally comparable models were computed for the ER in incongruent trials and the RT-Diff between congruent and incongruent trials of the Stroop task and the ER and RT of the go/no-go task. Each outcome is represented by two observed variables, each aggregating the scores (ER, RT, RT-Diff) for the first or second block, respectively. In all models, variance of the *i*th observed variable measured at occasion *t* of person *n* (${Y}_{itn}$) is decomposed into a within-person component (within-level) and a between-person component (between-level). At the within-level, the observed variables ${Y}_{w1tn}$ and ${Y}_{w2tn}$ are further decomposed into shared occasion factors ${OCC}_{tn}$, the linear effect of *time* with regression weight $\beta$, and the measurement error ${\epsilon }_{itn}$. There are autoregressive effects between ${OCC}_{tn}$ variables with fixed slope across persons $(\varphi )$. The ${OCC}_{tn}$ variables comprise the state residual variables as defined in LST-R theory but are not identical. In fact, the residuals of the autoregression correspond to the state residuals as defined in LST-R theory (for details see Eid et al., 2017; Stadtbaeumer et al., 2024). At the between-level, the outcomes ${Y}_{b1tn}$ and ${Y}_{b2tn}$, measure ${\xi }_{1n}$, the person-specific trait variable at *t*=1. The model allows for changes in traits, which means that outcomes ${Y}_{b1tn}$ and ${Y}_{b2tn}$ for *t* = 2, …, *T* may measure ${\xi }_{1n}$ plus trait changes due to the linear time trend and autoregression. The resulting model equations are as follows:

$$\begin{array}{ll}\text{Within-level}&Y_{w1tn}={1\cdot OCC}_{tn}+\beta\cdot time_t+\epsilon_{1tn}\\&Y_{w2tn}={1\cdot OCC}_{tn}+\beta\cdot time_t+\epsilon_{2tn}\\&{OCC}_{tn}=\varphi\cdot{OCC}_{t-1,n}+\zeta_{tn}\text{ for }t\hspace{0.17em}=\hspace{0.17em}2,\dots,T\\\text{Between-level }&Y_{b1tn}={1\cdot\xi}_{1n}\\&Y_{b2tn}={1\cdot\xi}_{1n}\end{array}$$

We assume equal variances for ${\zeta }_{tn}$-variables and ${\epsilon }_{itn}$-variables. The mean of ${\xi }_{1n}$ is freely estimated. The combined general equations correspond to

$$\begin{array}{c}{Y}_{1tn}={ Y}_{b1tn}+{ Y}_{w1tn}={\xi }_{1n}+{OCC}_{tn}+\beta\cdot tim{e}_{t}+{\epsilon }_{1tn}\\ { Y}_{2tn}={ Y}_{b2tn}+{ Y}_{w2tn}={\xi }_{1n}+{OCC}_{tn}+\beta\cdot tim{e}_{t}+{\epsilon }_{2tn}\end{array}$$

Considering the autoregressive structure with ${OCC}_{1n}={\zeta }_{1n}$ for the first time point and ${OCC}_{tn}=\varphi \cdot{OCC}_{t-1,n}+{\zeta }_{tn}$ for subsequent time points, this results in the full model equation for *t* = 1, …,*T* time points for any of the outcomes:

$${Y}_{itn}={\xi }_{1n}+\beta\cdot tim{e}_{t}+\sum_{j=1}^{t}{\varphi }^{j-1}\cdot{\zeta }_{jn}+{\epsilon }_{itn}$$

**Fig. 2:**
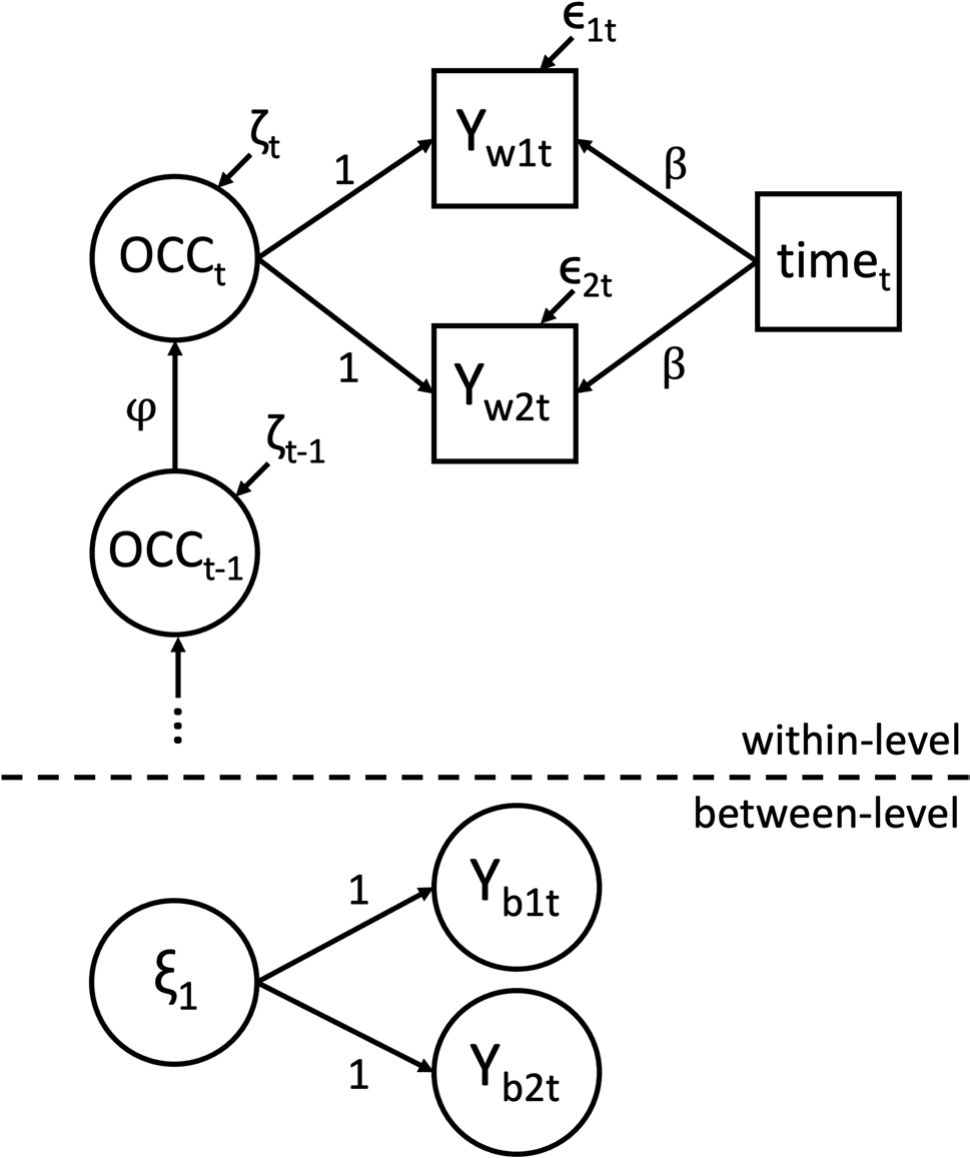
Model diagram of the dynamic structural equation models with autoregressive structure [AR(1) DSEM]. *Note.* The upper part represents within-person associations for a time point *t* and the previous time point *t* − 1, with within-level components of the dependent variable, Y_w1t_ and Y_w2t_, decomposed into time-specific occasion factors OCC and the effect of time. The occasion factors OCC_t_ and OCC_t-1_ with coefficient φ represent the autoregressive structure. The lower part represents between-person associations, with between-level components of the dependent variable, Y_b1t_ and Y_b2t_, and person-specific trait variable at time point 1, ξ_1_. To facilitate readability, the person-subscript *n* is omitted

Figure 2 illustrates the model conceptually.

Due to the autoregressive structure, the variance in the observed variable, Var(${Y}_{itn}$), comprises variance in ${\xi }_{1n}$, the residual variance of ${\epsilon }_{itn}$, and occasion-specific variance stemming from the state residuals. Assuming a stable autoregressive effect, we approximated Var(${Y}_{itn}$) by incorporating variance components from the previous four measurement occasions:

$$\begin{array}{l}Var\left({Y}_{itn}\right)=\text{Var}\left({\xi }_{1n}\right)+\text{Var}\left({OCC}_{tn}\right)+\text{Var}\left({\epsilon }_{itn}\right)\text{ with}\\ Var\left({OCC}_{tn}\right)\approx {\varphi }^{8}\cdot\text{Var}\left({\zeta }_{t-4,n}\right)+{\varphi }^{6}\cdot\text{Var}\left({\zeta }_{t-3,n}\right)+{\varphi }^{4}\cdot\text{Var}\left({\zeta }_{t-2,n}\right)+{\varphi }^{2}\cdot\text{Var}\left({\zeta }_{t-1,n}\right)+\text{Var}\left({\zeta }_{tn}\right)\end{array}$$

The within-person reliability is then defined as the proportion of systematic within-subject variance. Due to the autoregressive effects, this includes state residual variance and variance components from the previous four measurements:

$${Rel}_{within}\left({Y}_{witn}\right)=\frac{Var\left({OCC}_{tn}\right)}{\text{Var}\left({Y}_{witn}\right)}$$

The within-person reliability hence measures how reliably both indicators ${Y}_{1tn}$ and ${Y}_{2tn}$ capture fluctuations in responses within persons across time. In comparison, (overall) reliability, occasion specificity, and consistency relate not only to the within-level variance, but also to the total variance $Var\left({Y}_{itn}\right),$ and are calculated as follows:

$$\begin{array}{c}Rel\left({Y}_{itn}\right)=\frac{Var\left({\xi }_{1n}\right)+Var\left({OCC}_{tn}\right)}{Var\left({Y}_{itn}\right)}\\ Spe\left({Y}_{itn}\right)=\frac{Var\left({\zeta }_{tn}\right)}{Var\left({Y}_{itn}\right)}\\ Con\left({Y}_{itn}\right)=\frac{Var\left({\xi }_{tn}\right)}{Var\left({Y}_{itn}\right)}\end{array}$$

Given a specific time point *t*, the linear time trend does not sum to the variance of ${Y}_{itn}$ because it is constant. By definition, it holds that $Rel\left({Y}_{itn}\right)=Spe\left({Y}_{itn}\right)+Con\left({Y}_{itn}\right)$. Note that occasion specificity, in contrast to the within-person reliability, quantifies the proportion of state residual variance in the total variance. This excludes the autoregressive effects and hence measures the influence of purely situational effects and person-situation interactions.

#### Association of within-subject reliability with test length (number of Stroop and go/no-go trials) and stability of within-subject reliability across the duration of the study

To determine the number of trials necessary to reach adequate levels of measurement reliability (i.e., agreement between the two blocks of each task), we first calculated the relevant task scores considering increasing numbers of trials per block. For example, we calculated the Stroop ER and RT-Diff when 2, 4, 6, ..., *n* trials (always 50% congruent and 50% incongruent trials) of a block were considered. For each number of trials, the within-subject reliability ${R}_{\text{c}}$ (i.e., reliability of change; similar to Sliwinski et al., 2018) was computed using the mlr function of the R package *psych* (Revelle, 2023) for the two blocks of the same cognitive task measured during the same measurement occasion:

$${R}_{\text{c}}=\frac{{\sigma }_{p\cdot t}^{2}}{{\sigma }_{p\cdot t}^{2}+\frac{{\sigma }_{\epsilon }^{2}}{m}}$$

where ${\sigma }_{p\cdot t}^{2}$ and ${\sigma }_{\epsilon }^{2}$ represent the variance components of the person by measurement occasion and the measurement error, respectively. The number of items (i.e., the two block scores) is denoted as $m$.

Also following Sliwinski et al. (2018), we examined the stability of the within-subject reliability across the ambulatory assessment period by estimating the within-subject reliability separately for each day of assessment.

#### Convergent validity

To test the convergent validity, between- and within-subject Pearson correlations were computed using the statsBy function of the R package *psych* (Revelle, 2023). Correlations between the scores of the Stroop and the go/no-go task and self-report measures were examined. The self-report measures were executive function problems (i.e., Webexec score), progress in goal pursuit, desire strength, conflict of a desire with one’s goals, resistance to a desire, conduciveness of the environment (i.e., involving situational strategies of self-control), and the sum score of the two items measuring self-discipline and willpower.

#### Total and within-subject variance explained by contextual characteristics

Finally, to investigate how much of the within-subject variance was explained by contextual variables, multilevel models were computed using the R package *lme4* (Bates et al., 2015). In separate models, the test scores were included as outcome variables. In each model, time of day (i.e., beep number), social situation, location, and tiredness were included as predictors. For this analysis, all continuous variables (task scores and tiredness) were first within-subject-centered and then grand-mean-standardized. For each model, we report the explained within-subject variance for the fixed effects based on Nakagawa et al. (2017).

## Results

### Descriptive statistics

**Fig. 3:**
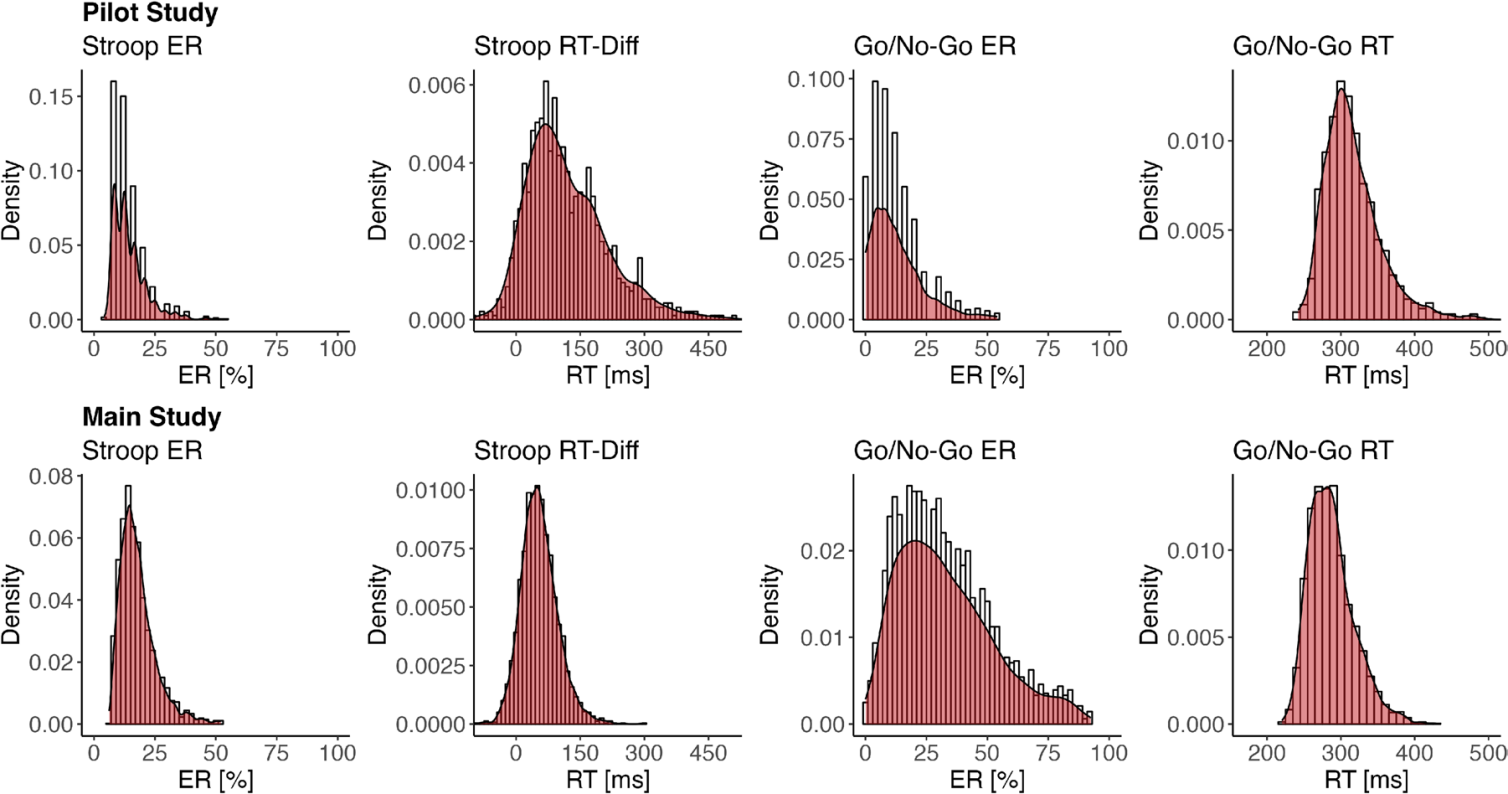
Distribution of task scores. *Note*. *ER* error rate; *RT* response time; *RT-Diff* difference in response times between congruent and incongruent trials

Demographics and descriptive statistics are displayed in Table 1. The distribution of task scores is displayed in Fig. 3.

**Table 1:** Demographics and descriptive statistics

	Pilot study (*N* = 21)	Main study (*N* = 70)
	*n* (%) / *M* (*SD*)	*n* (%) / *M* (*SD*)
Demographics		
Gender		
Female	14 (66.7)	48 (68.6)
Male	7 (33.3)	18 (25.7)
No response	0 (0.0)	4 (5.7)
Age (years)	25.1 (3.4)	24.7 (3.4)
Ambulatory assessment		
Average total duration^a^ [min]	6.7 (2.4)	15.5 (4.0)
Stroop		
Complete measurement occasions [*n*]	47.1 (21.4)	35.6 (18.3)
Completion rate [%]	67.4 (30.5)	50.9 (26.1)
Valid measurement occasions [*n*]	46.8 (21.2)	34.8 (18.3)
Excluded measurement occasions [*n*]	0.3 (0.8)	0.8 (2.1)
Average task duration [min]	2.0 (0.6)	4.0 (3.9)
ER [%]	14.6 (4.1)	19.1 (5.9)
RT-Diff [ms]	137.5 (70.0)	55.8 (35.8)
Go/no-go		
Complete measurement occasions [*n*]	46.9 (21.5)	35.3 (18.4)
Completion rate [%]	67.0 (30.8)	50.5 (26.3)
Valid measurement occasions [*n*]	46.5 (21.4)	34.5 (18.4)
Excluded measurement occasions [*n*]	0.4 (1.0)	0.8 (2.0)
Average task duration [min]	1.8 (0.3)	4.3 (1.8)
ER [%]	13.1 (6.3)	34.3 (15.9)
RT [ms]	319.1 (28.0)	287.6 (20.5)
Self-report		
Webexec score	148.3 (92.1)	152.9 (94.8)
Goal progress	63.1 (15.8)	62.3 (18.5)
Desire strength	62.7 (11.6)	58.9 (17.1)
Desire resistance	49.9 (19.8)	50.0 (21.3)
Environment adjustment	50.7 (22.1)	42.9 (22.9)
Self-discipline/willpower	104.7 (31.6)	117.7 (34.7)
Feeling tired	45.2 (14.1)	47.5 (19.9)
Being outdoors [*n*]	3.0 (3.1)	3.4 (4.3)
Being alone [*n*]	23.2 (12.5)	16.6 (11.9)

*Note*. The values for the ambulatory assessment variables represent the averages mean values per subject

*ER* error rate; *RT* response time; *RT-Diff* difference in response times between congruent and incongruent trials

^a^Including self-report, Stroop task, go/no-go task, and fruit tapping task

**Fig. 4:**
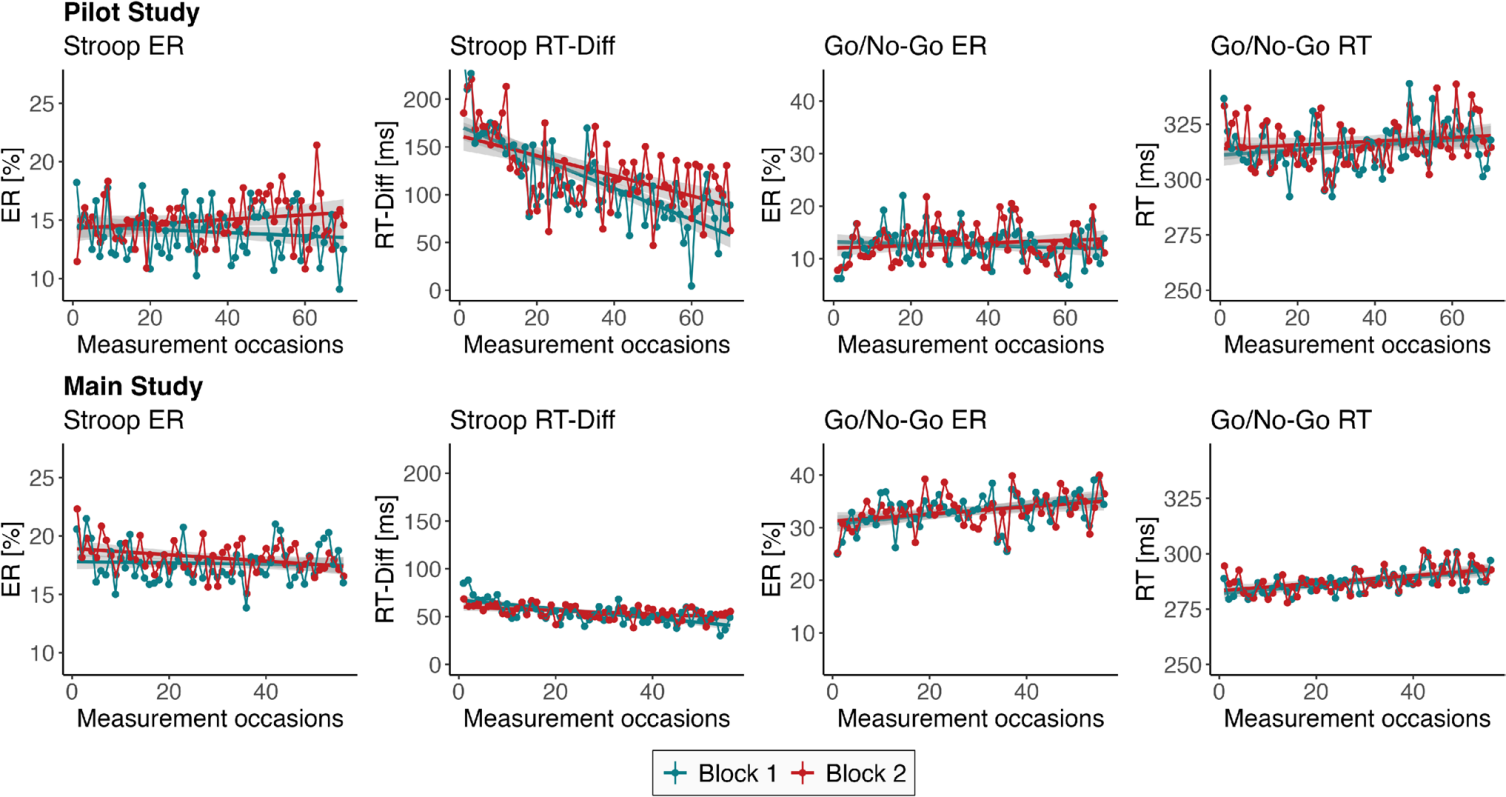
Trend of task scores across the data collection period. *Note*. *ER* error rate; *RT* response time; *RT-Diff* difference in response times between congruent and incongruent trials

In Fig. 4, averaged task measures are displayed across the whole study period. The time trend over the 14 days of measurement indicate a learning curve for Stroop task RT-Diff, with RT-Diff decreasing by about 50% across the 14 days in the pilot study, while at the same time no increase in Stroop ERs took place. In the main study, the decrease in RT-Diff appeared to be much smaller. Go/no-go RTs and ERs increased slightly across the 14 days in the main study.

### (Overall) reliability, common consistency, occasion specificity, and within-subject reliability

All parameters of interest resulting from the DSEM models for both studies are displayed in Table 2. In the pilot study and the main study, the Stroop ER total variance included 50.3% and 62.7% systematic variance, respectively (i.e., indicating reliability of .503 and .627), which can be dissected into 39.0% and 51.3% explained by common consistency (i.e., based on trait-level variance) and 10.8% and 11.1% explained by occasion specificity (i.e., based on state residual variance), respectively (all indices on scale level, that is, both blocks of the respective tasks combined). The within-subject reliability was .192 and .297, respectively. The Stroop task RT-Diff total variance included 54.6% and 40.7% systematic variance (i.e., indicating reliability of .546 and .407); 48.2% and 37.6% were accounted for by common consistency and 2.3% and 1.4% by occasion specificity in the pilot study and the main study, respectively. The within-subject reliability was .123 and .081, respectively.

**Table 2:** Coefficients as estimated by the dynamic structural equation model with a linear trend

Task	Score	Study	(Overall) reliability	Common consistency	Occasion specificity	Within-subject reliability
Stroop	ER	Pilot	.503	.390	.108	.192
		Main	.627	.513	.111	.315
	RT-Diff	Pilot	.546	.482	.023	.123
		Main	.407	.376	.014	.081
Go/no-go	ER	Pilot	.690	.393	.294	.534
		Main	.846	.685	.161	.576
	RT	Pilot	.863	.555	.307	.712
		Main	.869	.497	.372	.763

*Note. ER* error rate; *RT* response time; *RT-Diff* difference in response times between congruent and incongruent trials

The go/no-go ER total variance included 69.0% and 84.6% systematic variance (i.e., indicating reliability of .690 and .846); 39.3% and 68.5% were accounted for by common consistency and 29.4% and 16.1% by occasion specificity in the pilot study and the main study, respectively. The within-subject reliability was .534 and .576, respectively. The go/no-go RT total variance included 86.3% and 86.9% systematic variance (i.e., indicating reliability of .863 and .869); 55.5% and 49.7% were accounted for by common consistency and 30.7% and 37.2% by occasion specificity in the pilot study and the main study, respectively. The within-subject reliability was .712 and .763, respectively.

### Association of within-subject reliability with test length (number of Stroop and go/no-go trials) per measurement occasion and stability of within-subject reliability across the duration of the study

**Fig. 5:**
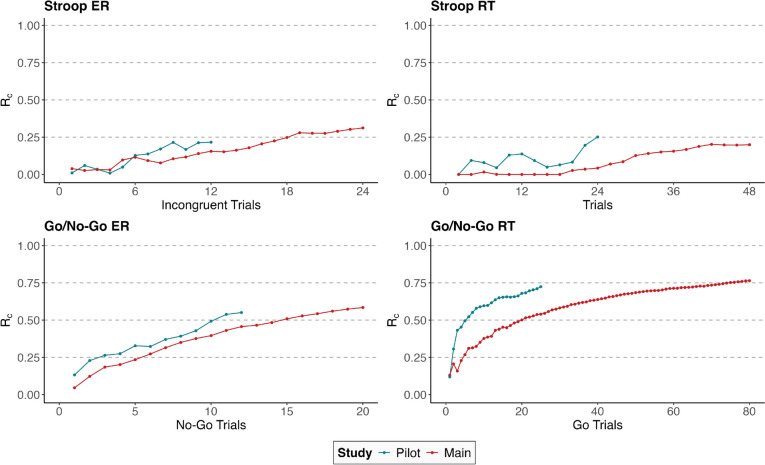
Within-subject reliability with increasing number of trials. *Note*. *ER* error rate; *RT* response time; *RT-Diff* difference in response times between congruent and incongruent trials; note that the *x*-axis displays the number of trials per block, which is half of the total trials. One block of the Stroop task consisted of 12 congruent and 12 incongruent trials in the pilot study and 24 congruent and 24 incongruent trials in the main study. One block of the go/no-go task consisted of 12 no-go and 28 go trials in the pilot study and 20 no-go and 80 go trials in the main study

Reliability–task-length plots are shown in Fig. 5 and indicate that the within-subject reliability increased with the number of trials. Whereas for the Stroop task, moderate levels of within-subject reliability were never reached, for the go/no-go ER, moderate levels of within-subject reliability were reached after 11 no-go trials in the pilot study and 15 no-go trials in the main study, and for the go/no-go RT, that level was reached after seven go trials in the pilot and 23 go trials in the main study. The settings of the go/no-go task in the pilot study (i.e., 30% of no-go trials in the pilot study vs. 20% of no-go trials in the main study) appeared to have helped in obtaining acceptable within-subject reliability within a shorter test duration considering the overall go/no-go task duration of 1.8 minutes in the pilot study versus 4.3 minutes in the main study.

**Fig. 6:**
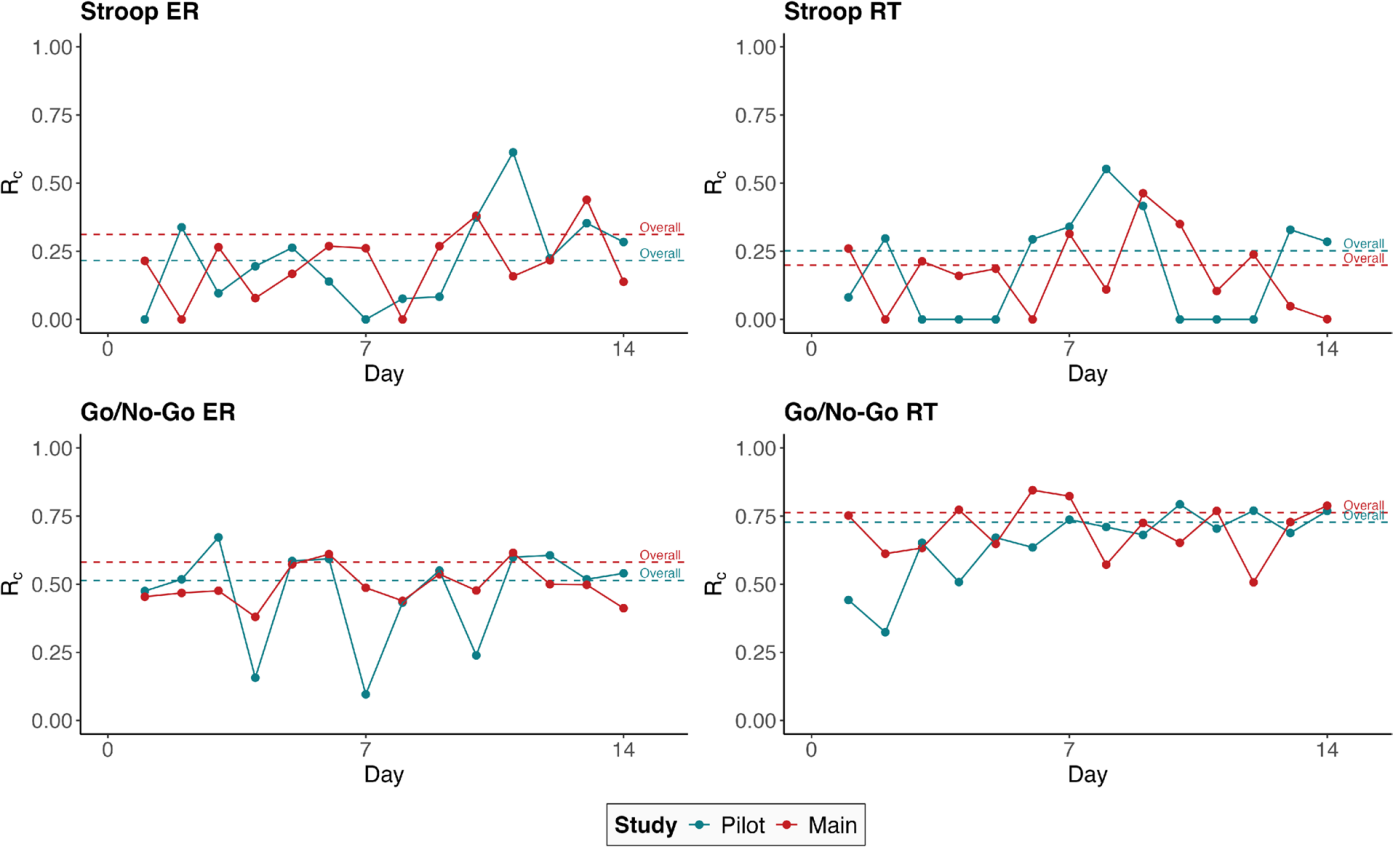
Within-subject reliability for each day of ambulatory assessment. *Note*. *ER* error rate; *RT* response time; *RT-Diff* difference in response times between congruent and incongruent trials

Figure 6 shows the within-subject reliability estimated separately for each of the 14 days of the EMA study. Across the 14 study days, within-subject reliability showed no systematic change that was consistent between pilot and main study for any of the indicators. In the main study in particular, the within-subject reliability of the go/no-go task ER and RT was at a consistently high level across the 14 days. On average, the within-subject reliability estimated separately for each of the 14 days was lower than the overall average within-subject reliability, which is attributed to a lower level of systematic within-subject variability within a given day as compared to systematic within-subject variability within and across days.

### Convergent validity

#### Within-subject level

Concerning convergent validity, as hypothesized, the Stroop ER was correlated with the go/no-go ER at the within-subject level in the pilot study (*r* = .08, *p* < .05) and in the main study (*r* = .12, *p* < .001). In the main study only, the Stroop ER was also correlated with the go/no-go RT (*r* = .07, *p* < .001) and the Stroop task RT-Diff was correlated with the go/no-go ER (*r* = −.07, *p* < .01).

**Fig. 7:**
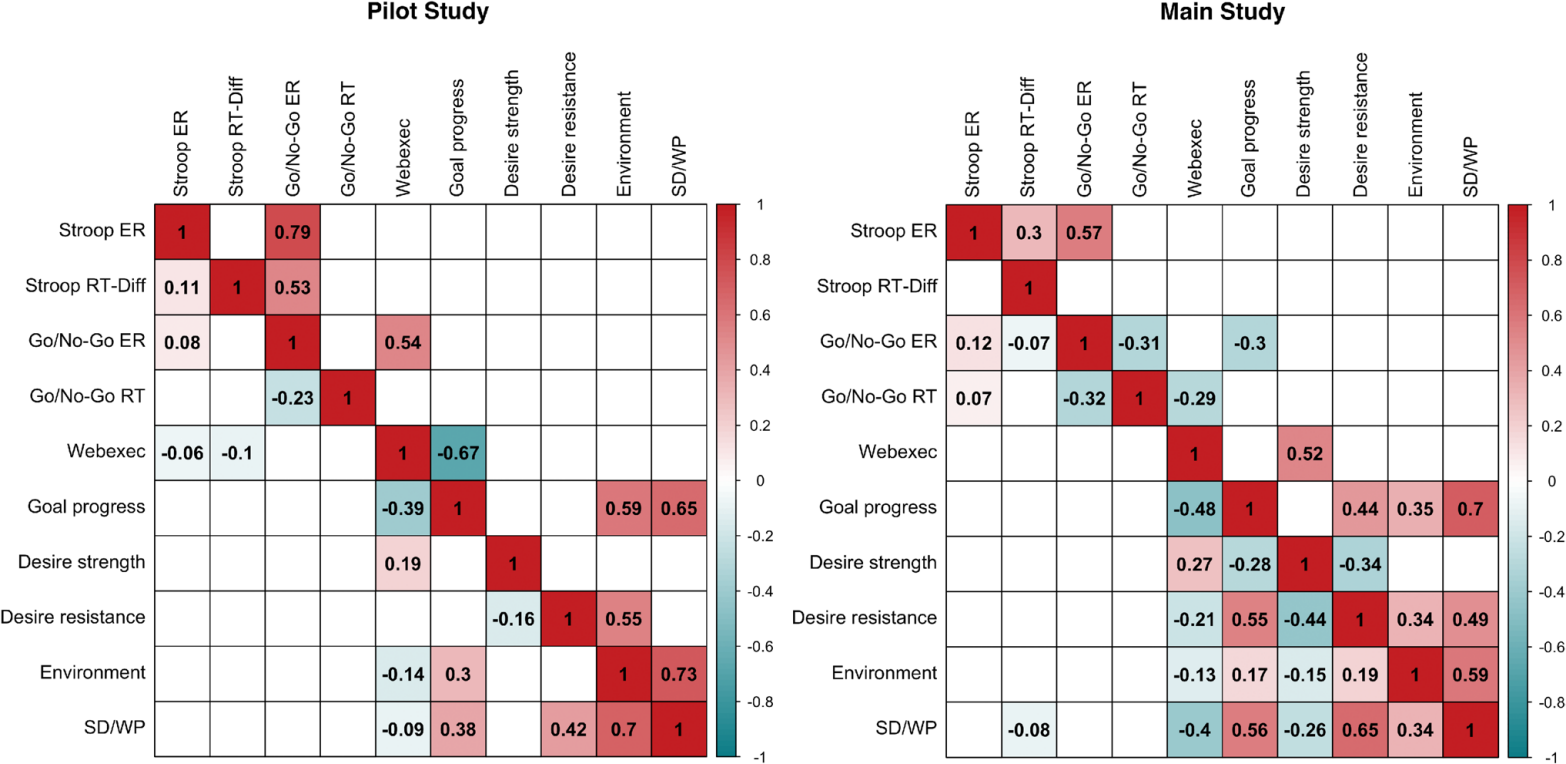
Correlation matrix for convergent validity. *Note*. *ER* error rate; *RT* response time; *RT-Diff* difference in response times between congruent and incongruent trials. Upper-right triangles display between-subject correlations and lower-left triangles display within-subject correlations. Only significant correlations are shown

Concerning the self-report measures, the Stroop ER (*r* = −.06, *p* < .05) and Stroop task RT-Diff (*r* = −.10, *p* < .01) were correlated with the Webexec score in the pilot study. In the main study, the Stroop task RT-Diff was also correlated with the self-discipline/willpower score (*r* = −.08, *p* < .05) in the main study. No other significant correlations between the task scores and self-report measures were found (Fig. 7).

#### Between-subject level

Concerning convergent validity, the Stroop ER was correlated with the go/no-go ER at the between-subject level in the pilot study (*r* = .79, *p* < .001) and in the main study (*r* = .57, *p* < .001). Also, the Stroop task RT-Diff was correlated with the go/no-go ER (*r* = .53, *p* < .05) in the pilot study.

Concerning the self-report measures, the go/no-go ER was correlated with the Webexec score at the between-subject level in the pilot study (*r* = .54, *p* < .05) but not in the main study. In the main study, however, the go/no-go ER was correlated with the progress in achieving one’s goals (*r* = −.30, *p* < .01) and the go/no-go RT was correlated with the Webexec score (*r* = −.29, *p* < .05).

### Variance explained by contextual characteristics

The contextual factors explained 6.0% and 3.4% of the Stroop ER variance in the pilot and main studies, respectively. Feeling more tired than usual (β = −0.05, *p* < .05) and being around other people (β = −0.13, *p* < .01) were associated with a lower Stroop ER in the main study.

The contextual factors explained 2.7% and 2.2% of the Stroop RT-Diff variance in the pilot and main studies, respectively. None of the contextual factors was significantly associated with Stroop RT-Diff in either study.

The contextual factors explained 6.4% and 4.0% of the go/no-go ER variance in the pilot and main studies, respectively. In the main study, the go/no-go ER differed among different times of the day. The go/no-go ER was significantly higher at beep 3 (β = 0.20, *p* < .01) and beep 4 (β = 0.14, *p* < .05) than at beep 1.

The contextual factors explained 13.9% and 5.2% of the go/no-go RT variance in the pilot and main studies, respectively. In the pilot study, we found differences in the go/no-go RT for different times of the day. The go/no-go RT was significantly lower at beep 3 (i.e., in the late afternoon; β = −.24, *p* < .05) than at beep 1 (i.e., in the morning).

## Discussion

### (Overall) reliability, common consistency, occasion specificity, and within-subject reliability

To the best of our knowledge, this study is the first to examine the within-subject reliability, occasion specificity, and validity of fluctuations of the Stroop task and the go/no-go task in an EMA context with multiple daily assessments over several days and applying LST-R models. Our findings indicate moderate overall reliability but poor within-subject reliability estimates for the Stroop task ER and Stroop task RT-Diff in both studies. For the go/no-go task ER and RTs, overall reliability was excellent and within-subject reliability was moderate to good in both studies. Thus, our findings indicate a moderately good ability of the go/no-go task when administered via EMA to reliably capture within-subject fluctuation of cognitive control. By contrast, the Stroop task as administered as in the present studies was not able to reliably capture moment-to-moment fluctuation of cognitive control. Instead, its moderate (overall) reliability was driven mainly by common consistency (i.e., trait-level variance).

Compared to a previous study estimating (overall) reliability, common consistency, and occasion specificity (Faßbender et al., 2023), it is notable that the amount of variance explained by occasion specificity was much larger and overall quite substantial in the current two studies, at least regarding the go/no-go task. This pattern did not appear for the Stroop task, where the occasion specificity of the Stroop task ER and RT-Diffs were at a very low level and similar to Faßbender et al.’s study. As the most important difference between Faßbender et al. (2023) and the current study, they studied state-level fluctuations only over three measurement time points using laboratory-based assessment, while the current two studies used an EMA design with a maximum of 70 measurement occasions in the pilot and 56 in the main study across 2 weeks and with smartphone-based assessment. Due to the increased number of measurement occasions and the more variable context and time of day of measurement, it is unsurprising that the two current studies found a larger amount of variance explained by state-level fluctuations at least with regard to the go/no-go task.

### Association of within-subject reliability with test length (number of Stroop and go/no-go trials) per measurement occasion and stability of within-subject reliability across the duration of the study

Increasing the number of test trials was associated with only a slight increase in the within-subject reliability of the Stroop task ER and RT. Reliable assessment of moment-to-moment fluctuations of cognitive control by further increasing the number of trials of the Stroop task does not appear to be practical, given that EMA exposes participants to considerable levels of burden, particularly when multiple daily measures are taken which run for several minutes per measurement occasion. Several observations suggest that the increased number of trials within the main study might have already decreased participants’ motivation: First, the completion rate decreased substantially from the pilot study to the main study. Second, a larger number of occasions had to be excluded in the main study, as criteria for careless responding were met. Third, the average Stroop ER was substantially increased in the main study compared to the pilot study, although the task itself was unchanged. These observations also apply to the go/no-go task in the main study as compared to the pilot study. However, the go/no-go task was changed slightly (i.e., the maximum allowed response time was decreased from 850 ms to 650 ms and the ratio of no-go trials to go trials was decreased from 30% no-go trials to only 20% no-go trials), which could at least partly explain the increase in the average error rate. With a focus on optimizing within-subject reliability while at the same time keeping subject burden as low as possible, the go/no-go task setup of the pilot study was preferable over the setup of the main study, as acceptable levels of within-subject reliability were obtained with a smaller number of trials and in a shorter length of time.

The within-subject reliability of the two tasks showed no systematic increase or decrease across the 14 study days in either study when within-subject reliability was calculated separately for each study day. In the main study in particular, the daily course of the within-subject reliability of the go/no-go task ER and RT across the 14 study days was relatively constant and at a high level, which was similar to the findings of Sliwinski et al. (2018) for spatial working memory and processing speed.

### Convergent validity

Regarding validity estimates, the only correlation that was observed consistently across the two studies concerned Stroop ER and go/no-go ER. As we expected, both scores were positively correlated. Given the poor within-subject reliability of the Stroop task ER and Stroop task RT-Diff, and only moderate within-subject reliability of the go/no-go task ER and go/no-go RTs, it is unsurprising that the Stroop task and go/no-go task scores do not correlate strongly at the within-subject level due to attenuation. At the between-subject level, the same positive correlation was also found in both studies, although it was much stronger there. These strong between-subject correlations between Stroop ER and go/no-go ER in both of our studies suggest a common underlying trait accounting specifically for the ER of both tasks. By contrast, the Stroop RT was not correlated with go/no-go RT in our studies, while previous cross-sectional research reported positive associations between the Stroop interference score and go/no-go RT (Lamm et al., 2006) and between Stroop RT and go/no-go RT (Morooka et al., 2012). Moreover, we found no association between the Stroop RT and go/no-go ER at either the within-subject or the between-subject level in either study, which is consistent with Faßbender et al. (2023) and the notion that the Stroop RT and go/no-go ER measure have different underlying constructs. Future research will also have to establish within-subject (i.e., situation-specific) convergent validity for the go/no-go task by correlating it with cognitive control tasks other than the Stroop task.

No associations of the Stroop task or the go/no-go task scores with self-reported measures of executive functions and self-control were found in either of the two studies. These findings mirror results from cross-sectional research showing no association between self-reported self-control and cognitive control (Eisenberg et al., 2019; Paap et al., 2020; Saunders et al., 2018). Thus, the findings of the present study confirm the distinction between cognitive control as measured by cognitive control tasks and that measured by self-reported self-control and cognitive control. Recent research suggests that self-reported self-control and cognitive control tasks assess different underlying processes (Saunders et al., 2018). While self-reports may capture subjective perceptions of self-control and progress on tasks, laboratory tasks may target specific cognitive mechanisms. In real life, goal progress is facilitated through the use of strategies that preventively decrease the extent of goal conflict (Duckworth et al., 2016). Moreover, for real-life goal progress, motivational processes may be more important than the level of cognitive control. If individuals are intrinsically motivated for a task, they may be less tempted by conflicting goals (Milyavskaya et al., 2015; Werner & Milyavskaya, 2019; Inzlicht et al., 2021). It must be noted, however, that associations might be stronger in samples with higher levels of cognitive control problems such as clinical samples with addictive behavior. Moreover, it also must be noted that at the between-subject level, statistical power was limited in particular in the pilot study. Between-subject correlations of around *r* = −.30 between go/no-go ER and goal progress and between go/no-go RT and self-reported executive function problems (i.e., the Webexec Score) might indicate reliable between-subject covariation between these variables, which could not be confirmed in the pilot study dataset due to limited statistical power.

### Variance explained by contextual variables

We found only a few associations of contextual variables including time of day, location (being indoors vs. outdoors), social context (being together with other people vs. alone), and self-reported tiredness with Stroop or go/no-go test performance that were consistent across the two studies. One result which we found consistently in both studies was that the Stroop ER was lower for participants who felt tired. It is possible that participants who indicated feeling tired in the questionnaire increased their efforts to compensate for tiredness. However, a priori, we would have rather expected the opposite direction of association. With regard to the environmental context, it is possible that a more nuanced assessment of environmental variables (e.g., noise level, glare, and social interference by interaction partners) would reveal stronger and more consistent associations. However, as environmental variables could bias the two test blocks within a measurement occasion into the same or opposite direction (e.g., an interaction partner could interfere during both test blocks of the measurement occasion or during only one), it is difficult to discern whether interference by these variables would increase or decrease the association between the two test blocks within a measurement occasion.

### Limitations

The following limitations of our two studies must be taken into consideration. First, both studies included predominantly female university students. It is possible that results would look different in other populations. Second, the compliance rates of around 67% in the pilot study and 51% in the main study were substantially lower than the normally reported compliance rate of around 79% in EMA research (Wrzus & Neubauer, 2023), which might be due to the longer EMA measurement occasions, which took about 6–7 minutes per occasion in total in the pilot study and 15–16 minutes in the main study. Third, the measurement of contextual variables such as noise level, glare, and social interference by interaction partners could have been more nuanced in order to exclude their interference effects which might be unrelated to potential psychological variables of interest such as stress levels but might bias the test performance. Fourth, our protocol involved EMA measurement occasions, which were timed during predefined time windows. It is possible that with other types of EMA protocols that are more adaptive to the psychological circumstances, different results might arise. For instance, if participants could trigger EMA assessments themselves during moments of stress, it might be possible to measure stress effects more closely after stressors were encountered. Future research will have to examine the feasibility of such designs related to EMA assessment of cognitive control. Fifth, we only analyzed the Stroop and the go/no-go task. It is possible that with the use of other cognitive control tasks, the convergent validity estimates would be higher. Findings by Faßbender et al. (2023), for instance, suggest that cognitive control is not a unitary construct but involves a two-factor structure with two only moderately correlated factors. In that study, the go/no-go task was associated with the main “response inhibition” factor while the Stroop task was associated with an “interference factor.” It is therefore possible that cognitive control tasks involving interfering stimuli (such as the Eriksen flanker task) would show stronger correlations with the Stroop task while tasks without interfering stimuli (such as the stop-signal task) would show stronger correlations with the go/no-go task. Sixth, a sample size of 70 may not have provided sufficient power to detect small between-subject correlations. However, the focus of this study was on the within-subject level. Sevenths, there is a potential circularity in the assessment of convergent validity, given that both the Stroop and go/no-go tasks were evaluated within the same study. This could lead to circular reasoning, where the validity of each task is inferred from its correlation with the other, despite both being under examination. Eighth, the within-subject reliability that was computed to investigate its association with test length and stability across the study duration differs from the reliability measures computed in DSEM. This discrepancy could lead to situations where the number of trials deemed sufficient for traditional reliability may not necessarily meet the reliability requirements as computed by DSEM. Finally, to estimate within-subject reliability and occasion specificity, we analyzed the common fluctuations of two test halves, while it would have been possible to conduct the analyses at the single trial level. Thus, future research might consider adding an additional level to the analysis by treating Stroop and go/no-go ERs and RTs on the trial level, with trials nested in measurement occasions, because it has been shown recently that this affects reliability (Rouder & Haaf, 2019; Waltmann et al., 2022; Zech et al., 2023).

## Conclusion

Using an LST-R model accounting for autoregression and trait change, we found moderately high within-subject reliability of the go/no-go task ER and RT as conducted on smartphones in the EMA context. By contrast, the Stroop task ER and RT did not reach at least borderline acceptable levels of within-subject reliability. The amount of occasion-specific variance was substantially larger for the go/no-go task in the EMA context than in laboratory research, but systematic state-level variance remained considerably smaller than systematic trait-level variance. Indicative of convergent validity, the within-subject correlations between the Stroop ER and go/no-go ER were positive and consistent across our two studies, but effect sizes were modest. Future research will have to test whether the findings generalize to more diverse social groups and are consistent with different cognitive control tasks, and will have to determine the role of interfering effects of the environmental context by using a more nuanced assessment of contextual variables.

## Data Availability

The data and materials used in this article are freely available on Open Science Framework (https://osf.io/rjbx2/).

## Code availability

The analysis code used in this article is freely available on the Open Science Framework (https://osf.io/rjbx2/).
